# Complications and outcomes in diffuse large B‐cell lymphoma with gastric lesions treated with R‐CHOP

**DOI:** 10.1002/cam4.1982

**Published:** 2019-02-07

**Authors:** Tomohiro Kadota, Sachiko Seo, Hiroe Fuse, Genichiro Ishii, Kuniaki Itoh, Tomonori Yano, Kazuhiro Kaneko, Kunihiro Tsukasaki

**Affiliations:** ^1^ Department of Gastroenterology, Endoscopy Division National Cancer Center Hospital East Kashiwa Japan; ^2^ Department of Hematology National Cancer Center Hospital East Kashiwa Japan; ^3^ Department of Hematology & Oncology Dokkyo Medical University Tochigi Japan; ^4^ Department of Hematology Matsudo City General Hospital Matsudo Chiba Japan; ^5^ Division of Pathology, Exploratory Oncology Research & Clinical Trial Center (EPOC) National Cancer Center Kashiwa Chiba Japan; ^6^ Division of Hematology/Oncology Kimitsu Chuo Hospital Kisarazu Japan; ^7^ Department of Hematology, International Medical Center Saitama Medical University Hidaka Japan

**Keywords:** bleeding, diffuse large B‐cell lymphoma with gastric lesions, gastric complications, mortality, R‐CHOP

## Abstract

Standard therapy for gastric diffuse large B‐cell lymphoma (DLBCL) is considered to be chemotherapy with or without involved‐field radiation therapy. Although R‐CHOP therapy alone is widely used for DLBCL with gastric lesions (DLBCL‐GL), the outcome and incidence of treatment‐related gastric complications following R‐CHOP are not well known. This study aimed to evaluate the outcome after R‐CHOP therapy in patients with gastric DLBCL including gastric complications and to identify risk factors for the complications. Consecutive patients with newly diagnosed DLBCL‐GL treated with R‐CHOP between 2003 and 2014 were retrospectively evaluated. DLBCL‐GL was defined only when pathologically confirmed in the stomach. Of the 96 patients with DLBCL‐GL, 63 patients were diagnosed with gastric symptoms. Eighty‐eight patients (92%) completed six to eight cycles of R‐CHOP. The complete remission (CR) rate was 86%, and 3‐year and 5‐year overall survival rates were 80% and 73%, respectively. Patients were well stratified according to the Revised International Prognostic Index (R‐IPI). Complication rate was 8% (8/96); seven patients had bleeding and three had stenosis. No patients had gastric perforation. Bleeding occurred during the first cycle of R‐CHOP in five patients (5/7, 71%). Patients with gastric complications had a lower R‐CHOP completion rate (50%, *P* = 0.001) and a lower CR rate (25%, *P* < 0.001) than those without complications. A low serum albumin level at diagnosis was the only risk factor identified for gastric complications (*P *=* *0.001) and six of the eight patients with complications were shown to be at stage IV. Further studies of DLBCL‐GL are warranted to identify patients at high risk for gastric complications and to provide better treatment strategies.

## 1 INTRODUCTION

Diffuse large B‐cell lymphoma (DLBCL) is a subtype of aggressive non‐Hodgkin lymphoma (NHL), accounting for 40%‐50% of all NHLs.^1^ Primary gastric DLBCL, which is usually defined as stage I or IIA according to the Lugano classification,^2^ is the most common extranodal DLBCL.^3^ However, gastric lesions are also sometimes observed in systemic DLBCL with or without gastric symptoms. After approval of the chimeric anti‐CD20 antibody, rituximab, R‐CHOP has been established as the first‐line treatment for DLBCL based on the results of several randomized phase III studies.^4^, ^5^ The current standard therapy for DLBCL with gastric lesions (DLBCL‐GL) is six to eight cycles of R‐CHOP or three cycles of R‐CHOP followed by involved‐field radiation therapy (IFRT) for early‐stage disease. Some reports showed the outcome of primary gastric DLBCL after R‐CHOP therapy^6^, ^7^, ^8^ while others reported that gastric complications such as bleeding, perforation, and stenosis, occurred under chemotherapy in 0%‐26%.^9^, ^10^, ^11^, ^12^, ^13^ However, the detailed features of these gastric complications were not well documented. Furthermore, there were few data on the outcomes of advanced DLBCL with gastric lesions.

This study aimed to evaluate outcomes in patients with DLBCL‐GL at all stages following R‐CHOP and the incidence of treatment‐related gastric complications. Moreover, we explored risk factors for gastric complications.

## 2 METHODS

### 2.1 Patients and study design

The present study enrolled patients who had pathologically confirmed DLBCL‐GL and were treated with R‐CHOP alone at the National Cancer Center Hospital East between October 2003 and July 2014. Patients who received the following procedures were excluded from the study: (a) surgical resection prior to chemotherapy, (b) radiotherapy, and (c) a chemotherapy regimen other than R‐CHOP alone. This study was approved by the institutional review board of the National Cancer Center (2015‐176).

### 2.2 Definitions

DLBCL in gastric lesion was identified based on pathological finding using tissue samples obtained by endoscopy. Clinical staging was determined according to the Ann Arbor classification,^14^ based on endoscopic findings, contrast‐enhanced computed tomography (CT) scan and/or positron emission tomography (PET)‐CT, and aspiration/biopsy of bone marrow. All patients were classified according to the International Prognostic Index (IPI)^15^ and Revised IPI (R‐IPI).^16^ Tumor response was determined 1 month after completion of the planned course of R‐CHOP (six to eight cycles) by the Revised Response Criteria for Malignant Lymphoma 2007.^17^ Gastric lesions were evaluated comprehensively by both endoscopic findings and biopsy to confirm complete remission (CR).

Gastric bleeding, stenosis, and perforation were defined as gastric complications. Gastric bleeding was defined as a bleeding episode requiring blood transfusion; stenosis was defined as the failure of ordinary endoscopy to pass through the pyloric ring. Gastric perforation was diagnosed when free air was shown to be present on abdominal x‐ray or CT scan.

### 2.3 Statistical analysis

All statistical analysis was performed using sex, age, serum lactate dehydrogenase (LDH) level, serum albumin level, clinical stage, and R‐IPI. Serum LDH and serum albumin before R‐CHOP were analyzed as continuous variables.

A univariate analysis was performed by applying the nonparametric Mann‐Whitney U test for serum LDH and serum albumin, and the Fisher's exact test for the other factors to assess the association between the clinical factors and gastric complications. Overall survival (OS) time was defined as the time from the start of chemotherapy to death from any cause. The probability of OS was estimated using the Kaplan‐Meier method, and the log‐rank test was used to compare hazards of time‐to‐event outcomes among risk groups. All variables were deemed to be significant if a two‐sided *P *value was* <*0.05. All data were analyzed using SPSS software (version 22.0 for Mac).

## 3 RESULTS

### 3.1 Patient characteristics

There were 109 patients who had pathologically confirmed DLBCL with gastric lesions. Of the 109 patients, 13 patients were excluded due to surgical resection prior to chemotherapy (n = 3), additional radiotherapy (n = 4), and CHOP without rituximab (n = 6). The patient characteristics at diagnosis are shown in Table 1. Gastric lesions were detected due to gastrointestinal symptoms in 63 patients (66%), gastric cancer screening by endoscopy in eight patients (8%), and clinical staging of lymphoma in 25 patients (26%). Gastrointestinal symptoms included abdominal pain (n = 42), loss of appetite (n = 9), bloody stool (n = 7), hematemesis (n = 3), and others (n = 2).

**Table 1:** Background characteristics of patients

	Total n (%)
Sex	
Men	58 (60)
Women	38 (40)
Age (y)	
median, range	68.5, 26‐85
Ann Arbor clinical stage	
I	32 (33)
II	20 (21)
III	6 (6)
IV	38 (40)
Opportunity of diagnosed gastric lesion	
Gastrointestinal symptom	69 (72)
Medical examination	8 (8)
Examination to investigate clinical stage	19 (20)
IPI	
Low	38 (40)
Low‐intermediate	25 (26)
High‐intermediate	13 (14)
High	20 (21)
R‐IPI	
Very good	10 (10)
Good	53 (55)
Poor	33 (34)
Serum LDH (IU/L)	
median, range	233, 127‐3980
Serum albumin (g/dL)	
median, range	3.6, 1.8‐4.7
Cycles of R‐CHOP	
8 cycles	47 (49)
7 cycles	3 (3)
6 cycles	38 (40)
Fewer than 6 cycles^a^	8 (8)

IPI, International prognostic index; R‐IPI, Revised International prognostic index.

a Including five cycles of R‐CHOP (n = 1), four cycles of R‐CHOP following one cycle of rituximab (n = 1), four cycles of R‐CHOP (n = 3), three cycles of R‐CHOP (n = 2), and one cycle of R‐CHOP (n = 1).

Eighty‐eight patients (92%) received six to eight cycles of R‐CHOP and eight patients (8%) received fewer than six cycles of R‐CHOP (Table 1). The reasons for failure to complete the planned R‐CHOP cycles were treatment‐related complications in five patients (bleeding in two, stenosis, cytopenia, and fatigue in one patient each) and disease progression in three patients.

### 3.2 Efficacy of R‐CHOP on gastric DLBCL

CR was achieved in 83 patients (86%). Among the patients completing six to eight cycles of R‐CHOP, the CR rate was 90% (79/88). As shown in Table 2, CR rates gradually reduced in more advanced stages or a higher‐risk group according to IPI or R‐IPI. The median follow‐up period was 48.8 months (range, 0.6 to 123.3 months). In a total of 14 patients, diseases recurred after confirmed CR (17%; 14/83).

**Table 2:** Complete Response rate according to clinical stage, IPI, R‐IPI, and number of cycles of R‐CHOP

	Complete response rate n (%)
Ann Arbor clinical stage	
I	31/32 (97)
II	18/20 (90)
III	5/6 (83)
IV	29/38 (76)
IPI	
Low	38/38 (100)
Low‐intermediate	21/25 (84)
High‐intermediate	12/13 (92)
High	12/20 (60)
R‐IPI	
Very good	10/10 (100)
Good	49/53 (92)
Poor	24/33 (73)
Cycles of R‐CHOP	
6‐8 cycles	79/88 (90)
Fewer than 6 cycles	4/8 (50)
Total	83/96 (86)

**Figure 1:**
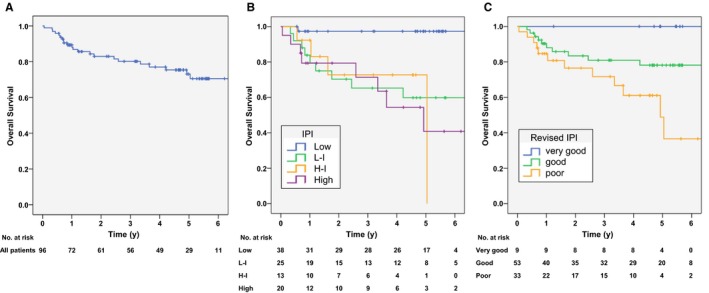
Overall survival (OS) curves according to International prognostic index (IPI), and revised IPI (R‐IPI). (A) OS in total patients. 3‐ and 5‐year OS rates were 80% and 73%, respectively. (B) OS curves according to IPI. The 5‐year OS rates were 97%, 60%, 73%, and 41% in low, low‐intermediate, high‐intermediate, and high‐risk groups, respectively (*P* < 0.001). (C) OS curves according to R‐IPI. The 3‐ and 5‐year OS rates were 100% and 100% in the “very good” category, 81% and 78% in the “good” category, and 72% and 49% in the “poor” category, respectively (*P* = 0.013)

The 3‐ and 5‐year OS rates were 80% and 73%, respectively (Figure 1A). Among the patients completing six to eight cycles of R‐CHOP, the 3‐ and 5‐year OS rates were 85% and 77%, respectively. The OS were poorly stratified by the Ann Arbor staging classification (*P = *0.002; Figure S1) or IPI (*P *< 0.001; Figure 1B), but well stratified by R‐IPI (*P = *0.013; Figure 1C).

### 3.3 Gastric complications

No patients had bleeding or stenosis that required endoscopic treatment prior to chemotherapy. After the initiation of chemotherapy, gastric complications occurred in eight patients (8%) (gastric bleeding, five; gastric stenosis, one; and gastric bleeding and stenosis, two). No patients had gastric perforation. The details were shown in Table 3.

**Table 3:** Cases with gastric bleeding and gastric stenosis

Case	Age/sex	Clinical stage	Serum albumin (g/dL)	Complication	Date of bleeding and stenosis (cycle and day)	Treatment for bleeding and stenosis	Follow‐up (duration from date of complication)
1	65 M	IV	3.1	bleeding	R‐CHOP 1 cycle, day20	Transcatheter embolization	Death of hemorrhagic shock (20 days)
2	58 M	II	3.3	bleeding	R‐CHOP1 cycle, day1	Conservative treatment	Death of DLBCL (10 months)
3	77F	IV	3.2	bleeding	R‐CHOP 1 cycle, day10	Endoscopic hemostasis	Palliative care
4	65 M	IV	2.1	bleeding	R‐CHOP1 cycle, day1	Total gastrectomy	Death of DLBCL (12 months)
5	73 M	IV	3.3	bleeding	R‐CHOP 8 cycle, day38	Endoscopic hemostasis	Death of DLBCL (11 months)
6	67 M	IV	3.3	bleeding	R‐CHOP 4 cycle, day22	Endoscopic hemostasis	Death of other diseases (12 months）
				stenosis	R‐CHOP 4 cycle	Surgical gastro jejunostomy	
7	76 M	IV	2.2	bleeding	R‐CHOP 1 cycle, day7	Conservative treatment	Death of DLBCL (31 months)
				stenosis	R‐CHOP 3 cycle	Endoscopic balloon dilation	
8	72F	I	2.7	stenosis	R‐CHOP 2 cycle	No treatment for stenosis	Death of DLBCL (5 months)

Of the seven patients with bleeding, five (71%) had bleeding during the first cycle of R‐CHOP (median, 15 days; range, 1‐206). Two were managed conservatively, while three required endoscopic hemostasis. However, two of these patients developed hemorrhagic shock following failure of endoscopic hemostasis; one was treated with total gastrectomy, while the other died of hemorrhagic shock despite transcatheter embolization. Among seven patients with bleeding, two had bleeding after multiple cycles of R‐CHOP and both patients had persistent lymphoma at the time of bleeding.

**Figure 2:**
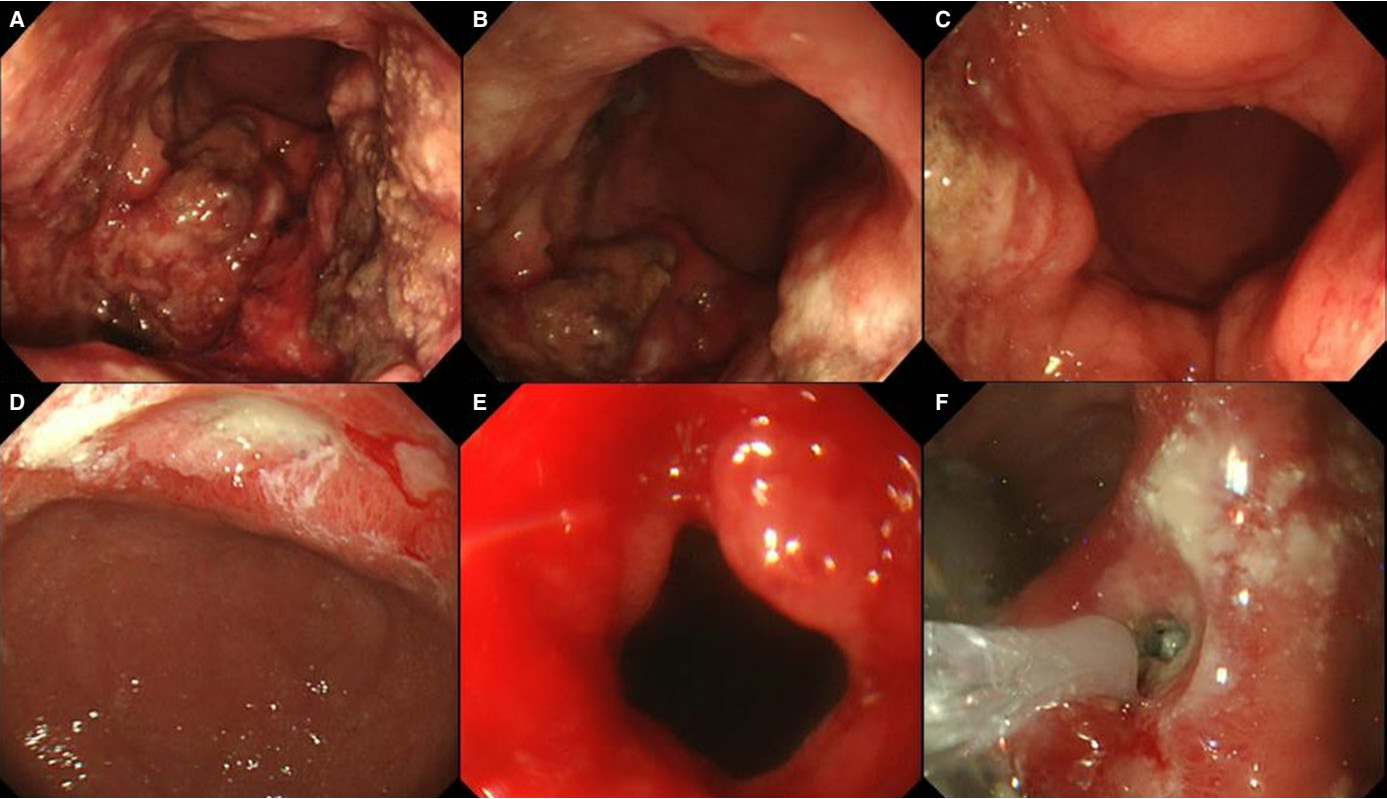
67‐year‐old woman who developed gastric stenosis during chemotherapy with R‐CHOP (case 6 in Table 3). (A) An ulcerative lesion was located mainly posterior to the gastric lower body at diagnosis. (B) The lesion was shown to have spread from the gastric lower body to the entire circumference of the antrum at diagnosis. (C) Ordinary endoscopy could pass the antrum and pyloric ring at diagnosis. (D) The ulcerative lesion improved after four cycles of R‐CHOP. (E) The ulcerative scar at the antrum caused stenosis and thus did not allow ordinary endoscopy to pass. (F) Although endoscopic balloon dilation was performed repeatedly, the stenosis did not improve

Gastric stenosis occurred in three patients between the second and the forth cycles of R‐CHOP. In all three patients, the lesion was shown to have spread through the entire circumference of the gastric antral zone before treatment. The stenosis was considered a consequence of post‐tumor scarring in two patients (#6 and 7) based on endoscopic findings, and surgical gastrojejunostomy and endoscopic balloon dilation were performed in one patient each. The stenosis in a patient (#8) was considered to be caused by residual disease, and no stenosis treatment was performed in the patient due to the patient's poor physical condition. Major endoscopic findings in a representative case (case 6 in Table 3) are shown in Figure 2.

### 3.4 Outcomes after gastric complications and risk factors

Of the eight patients with gastric complications, four patients (two with both bleeding and stenosis, one with bleeding, and one with gastric stenosis) did not receive six cycles of R‐CHOP due to complications or disease progression. Of these eight patients, six (75%) failed to achieve CR and six (five in non‐CR cases and one in CR cases) (75%) died from DLBCL. The completion rate for six cycles of R‐CHOP and CR rate were significantly lower among those with gastric complications than those without (completion rate, 50% (4/8) vs. 95% (84/88), *P* = 0.001; CR rate, 25% (4/8) vs. 92% (81/88), *P* < 0.001).

A comparison between patients with gastric complications and those without revealed that the serum albumin level was significantly correlated with the occurrence of complications (*P* = 0.001) (Table 4). No patients had gastric complications when their albumin levels were more than 3.4 g/dL (gastric complications:>3.4 of albumin, 0/61 patients; ≤ 3.4 of albumin, 8/35 patients; *P* < 0.001).

**Table 4:** Comparison between patients with and without gastric complications

	Patients with complication	Patients without complication	Univariate analysis
	(Total N = 8)	(Total N = 88)	*P‐*value
Sex			0.71
Men	4	54	
Women	4	34	
Age			0.71
≤70	4	55	
>70	4	33	
Serum LDH (median, range) [IU/L]	479.5, 147‐2890	226.5, 127‐3980	0.063
Serum albumin (median, range) [g/dL]	3.15, 2.1‐3.3	3.7, 1.8‐4.7	0.001
Ann Arbor clinical stage			0.055
I, II, III	3	53	
IV	5	33	
R‐IPI			0.12
Very good, Good	3	60	
Poor	5	28	

R‐IPI, Revised International prognostic index.

## 4 DISCUSSION

In our study, 86% of the 96 DLBCL‐GL patients who completed six to eight cycles of R‐CHOP achieved CR, and their 5‐year OS was 73%. The eight patients (8%), who developed gastric complications after R‐CHOP, had a significantly lower rate of treatment completion, resulting in a low CR rate. A low level of serum albumin before treatment was associated with the occurrence of gastric complications.

R‐CHOP is currently the first‐line therapy of choice for DLBCL. Although the efficacy of R‐CHOP on primary gastric DLBCL remains controversial,^6^, ^7^, ^8^, ^18^, ^19^ R‐CHOP is practically used for gastric DLBCL as well as for other DLBCL. Reported CR rates range between 92.5% and 100% and the 3‐year OS rates range between 84.7% and 100%.^6^, ^7^, ^18^, ^19^ In our study, the CR rate was 86% and the 3‐year OS rate was 80%. Although our results appear to be worse, 46% of our patients had Ann Arbor stage III/IV disease in contrast to only approximately 25% of patients having advanced disease in other studies that focused on primary gastric DLBCL (Lugano stage IIE/IV; 24%‐25%).^6^, ^19^ One important finding in our study is that patients with gastric complications after R‐CHOP tended to have advanced disease (six of eight patients with gastric complications) and discontinue treatment after occurrence of complications, resulting in low CR rates. Thus, completion of R‐CHOP therapy appears to be required to achieve better remission and survival in DLBCL‐GL.

Our study showed that R‐IPI stratified the prognosis in patients with DLBCL‐GL better than IPI or Ann Arbor staging. Although previous studies have reported poor stratification of DLBCL‐GL by IPI,^13^, ^19^ little is known about the utility of R‐IPI in DLBCL‐GL. R‐IPI was originally proposed as a better predictor of survival for patients with DLBCL in the era of R‐CHOP.^16^ In accordance with the results in DLBCL, our results also showed the superiority of R‐IPI to IPI in patients with DLBCL‐GL, which might be related to that of our cohort included a large proportion of advanced stage. Therefore, R‐IPI also appears to be used to predict the prognosis for patients with DLBCL‐GL although validation in another cohort is required.

There are a few reports describing the features of gastric complications occurring with chemotherapy, and gastric perforation and bleeding were considered as the result of rapid tumor necrosis by response to chemotherapy.^10^, ^12^ Bleeding occurred relatively early, usually around 10‐14 days after the first chemotherapy course.^10^ Similar to these previous studies, most bleeding (71%) occurred during the first cycle of R‐CHOP in our study. Since gastric bleeding after chemotherapy can be fatal as shown in previous studies and ours, careful observation is needed during the first cycle of R‐CHOP. Importantly, based on our results, strong caution for perforation after R‐CHOP may not be required for DLBCL‐GL. Although we have not evaluated the relation between tumor size and incidence of bleeding or perforation, tumor size can be a predictive marker for bleeding, and bleeding may be associated with disease persistence in some cases. Some groups have reported that endoscopic ultrasonography accurately estimates the depth of tumor infiltration in gastric lymphoma and can be used to help assess the risk of perforation or bleeding during chemotherapy.^20^, ^21^ Since no definite criteria for the risk estimation exist, routine evaluation by endoscopic ultrasonography is not currently performed.^13^ A prospective study is warranted to establish new useful endoscopic ultrasonography for identification of high‐risk patients.

Gastric stenosis occurred in three patients (3%) of our cohort during the two to four cycles of R‐CHOP, which was relatively late, suggesting the result of healing, scarring, and fibrosis at the site of the initial tumor.^12^ In this study, all three patients had gastric antral involvement at the time of diagnosis, which may be correlated with gastric stenosis. In cases of gastric cancer, gastroduodenal stent placement or surgical gastrojejunostomy was performed for gastric stenosis, so that most patients could receive chemotherapy.^22^, ^23^, ^24^ Although gastrectomy or conservative treatment was performed for complications following R‐CHOP, very few patients were able to continue chemotherapy in our study. In order to improve their completion of R‐CHOP and CR rates, gastrectomy or conservative treatment for gastric stenosis prior to R‐CHOP therapy appears to be required in gastric DLBCL patients with stenosis, as well as in patients with gastric cancer. In terms of deep ulceration or transmural infiltration, some reports have suggested that surgery may be primarily indicated for localized gastric DLBCL to avoid gastric complications.^25^

An intriguing finding in this study was that low serum albumin was a risk factor in DLBCL‐GL for gastric complications that were associated with worse survival. This correlation between serum albumin and gastric complications may indicate that the serum albumin level is a surrogate marker for severity of lymphoma in the stomach. Low serum albumin has been reported as a poor prognostic factor in aggressive lymphomas.^26^, ^27^ In DLBCL‐GL, the reduction in serum albumin may be due to lymphoma‐associated catabolic inflammation or to loss of albumin after gastric bleeding.

As a single‐institutional retrospective study, this study has several limitations. First, since there were only eight patients with gastric complications, we could not perform a multivariable analysis on the risk factors for these complications, despite being one of the largest studies involving patients with gastric DLBCL. Second, there were no definite criteria for the discontinuation of R‐CHOP. Physician discretion and patient choice may have affected the completion rate in the study. Third, precise information about bleeding before treatment was not available. Therefore, we were not able to analyze whether bleeding at diagnosis was a risk factor for gastric complications.

In conclusion, R‐CHOP as the first‐line therapy was effective in gastric DLBCL even in patients with advanced DLBCL‐GL, with 8% of patients developing gastric complications. Careful follow‐up is needed during chemotherapy in patients with advanced DLBCL‐GL, especially those with low serum albumin before treatment. Further studies for DLBCL‐GL are warranted to identify patients at high risk for developing gastric complications and to provide better treatment strategies.

## CONFLICTS OF INTEREST

K. Tsukasaki has received research funding from Celgene, Novartis Pharma, Phyzer and Chugai, and honoraria from KyowaKirin Hakkou, Chugai and Glaxo Smith Kline, and provided consultancy for Takeda Bio, Symbio, and Ono Pharma. K. Itoh has received research funding from Zenyaku kogyo Co. Ltd.
