# Supplementary figures and images for: Complications and outcomes in diffuse large B‐cell lymphoma with gastric lesions treated with R‐CHOP
Source: Cancer Med. 2019 Feb 7;8(3):982–9. doi: 10.1002/cam4.1982 (PMC6434211; doi:10.1002/cam4.1982)

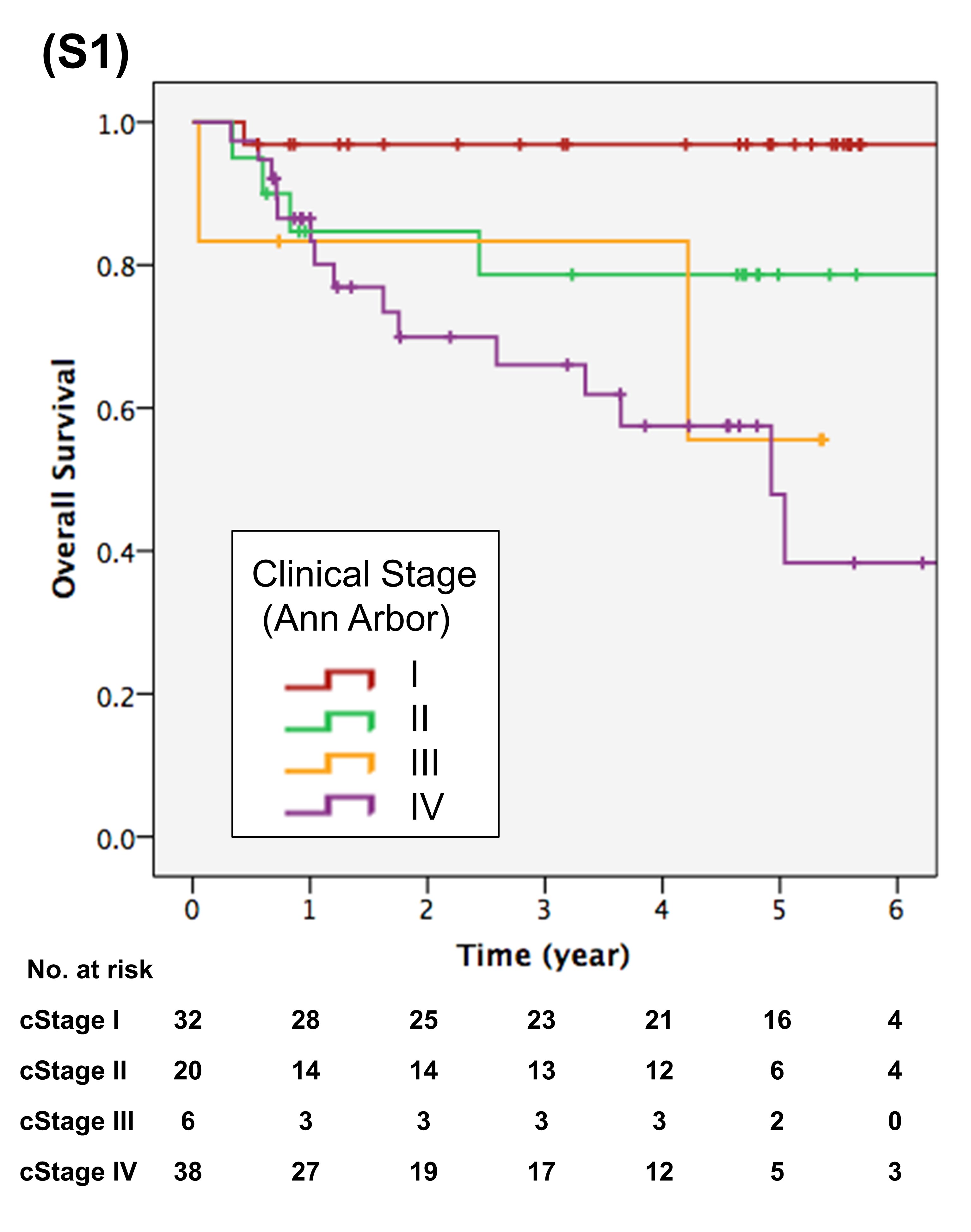

Supplement: Supplementary file 1 [file CAM4-8-982-s001.tif]
